# Largely different contents of terpenoids in beef red-flesh tangerine and its wild type

**DOI:** 10.1186/s12870-017-0988-4

**Published:** 2017-02-03

**Authors:** Wenyun Li, Cuihua Liu, Min He, Jinqiang Li, Yongqiang Cai, Yuhua Ma, Juan Xu

**Affiliations:** 1 Key Laboratory of Horticultural Plant Biology (Ministry of Education), College of Horticulture and Forestry, Huazhong Agricultural University, Wuhan, 430070 China; 2 Guizhou Fruit Institute,Guizhou Academy of Agricultural Sciences, Guiyang, Guizhou Province 550006 China; 3 College of Horticulture, Northwest A & F University, Yangling, Shanxi Province 712100 China

**Keywords:** *Citrus reticulate* Blanco, Niurouhong, Terpenoids, Volatile, Carotenoids, Phytohormone, Limonoid aglycones

## Abstract

**Background:**

Niurouhong (*Citrus reticulata* Blanco. Niurouhong) (NRH) is a spontaneous beef-red flesh mutant with distinctive flavor compared with its wild type orange-red flesh Zhuhongju (ZHJ). To illustrate the biochemical mechanism of its special flesh color and flavor, fruits at commercial mature stage were used to profile the volatiles in the flavedo and determine the levels of carotenoids, limonoid aglycones and phytohormones in the juice sacs in two seasons.

**Results:**

Our results showed the content of total volatile terpenoids in NRH was 1.27-fold that in ZHJ. The components of volatiles were found to be common between the two tangerines. This result indicates that the distinctive flavor of NRH might not be derived from the presence/absence of specific volatiles; instead, it was derived from the altered concentrations or balance of *α*-citral, *β*-citral, 2-cyclohexen-1-one, (S)-3-methyl-6-(1-methylethenyl) and n-hexadecanoic acid. Analyses of the contents of total and specific carotenoids indicated that the beef-red color of NRH flesh might be largely attributed to the over accumulation of *β*-cryptoxanthin and *β*-carotene. However, lower ABA level was found in NRH than in ZHJ, reflecting a possible feedback regulation of ABA biosynthesis on carotenogenesis and the balance in the metabolism among terpenoids.

**Conclusions:**

Collectively, our study suggested that the MEP pathway was enhanced in NRH tangerine. However, a certain unknown co-regulatory mechanism might be present in the metabolism pathway of secondary metabolites (especially terpenoids) in beef-red flesh mutant. Our study provides new insights into the regulatory network of terpenoid metabolism and mutation mechanism of red-fleshed citrus.

## Background

Plant terpenoids are important metabolites, and represent a class of hydrocarbons that have functions in photosynthesis, respiration and regulation of plant growth and development. They also have substantial economic values to serve as essential oils, colorants, flavors and anti-oxidative drugs [1]. All terpenoids are derived from the condensation of two common 5-carbon precursors in MEP and/or MVA pathway to produce monoterpenes (C10), sesquiterpenes (C15) and diterpenes (C20) under the catalysis of various terpene synthases (TPSs) [2]. In *Arabidopsis*, TPS21 and TPS11 are responsible for the biosynthesis of nearly all 20 sesquiterpenes [3]. Citrus plants are rich of terpenoids, and volatile organic compounds, phytohormones, carotenoids, limonoid aglycones are produced in leaves, flowers, roots and fruits. Monoterpenes and sesquiterpenes account for more than 80% of the total volatiles in citrus (Yamamoto et al. [4]), and carotenoids (C_30_ and C_40_) and limonoid aglycones are the derivatives of tetraterpenoids and triterpenoids [4], while ABA is regarded as a sesquiterpene usually produced from the degradation of carotenoids [5]. Terpenoids in citrus play key roles in photosynthesis, plant growth regulation, plant-environment interactions and fruit quality.

Most citrus fruits are generally colored by yellow or orange carotenoids particularly xanthophylls in the plastids during maturation [6]. Some red-fleshed citrus cultivars, such as “Cara Cara” navel orange (*C. sinensis* Osbeck) [7] and and red-fleshed pummelo (*C. grandis* Osbeck) predominantly accumulate lycopene and *β*-carotene [8], whereas blood orange (*C. sinensis* Osbeck) is characterized by the accumulation of anthocyanin [9]. Red-fleshed fruit is not only special in flesh color, but also distinctive in other qualities compared with wild-type fruit. Chen et al. [10] reported that the contents of some flavonoids are significantly different between red-fleshed and blonde-fleshed sweet oranges. Yoo et al. [11] revealed the differences in the contents of ascorbic acid, sugar, soluble solid and total carotenoid among 11 watermelons with different pulp colors, while different carotenoid, limonoid and aroma profiles of two pummelos (*Citrus* maxima) with different flesh color were also reported in citrus by Liu et al. [12].

Tangerine (*C. reticulata* Blanco) is well known as one of the four main cultivated citrus species for its wide application in juice industry. Its fresh fruits can be processed or marketed owing to the traits of easy peeling, appealing color, pleasant aroma and good taste. Tangerine fruit quality is usually determined by several characters such as color, aroma and bitterness [13]. A great part of these characters are determined by the composition of terpenoids, which constitute the largest and most diverse class of secondary or specialized metabolites mainly represented by phytohormones of abscisic acid (ABA) and gibberellic acid (GA), carotenoids, volatile terpenoids and bitterness compounds of limonoid aglycones in Citrus species [14]. Citrus fruit color is mainly derived from the accumulation of carotenoids while flavonoids contribute to the yellow background color. Its aroma is mainly derived from volatile organic compounds (VOCs), whereas delayed bitterness usually results from the accumulation of limonin.

**Fig. 1:**
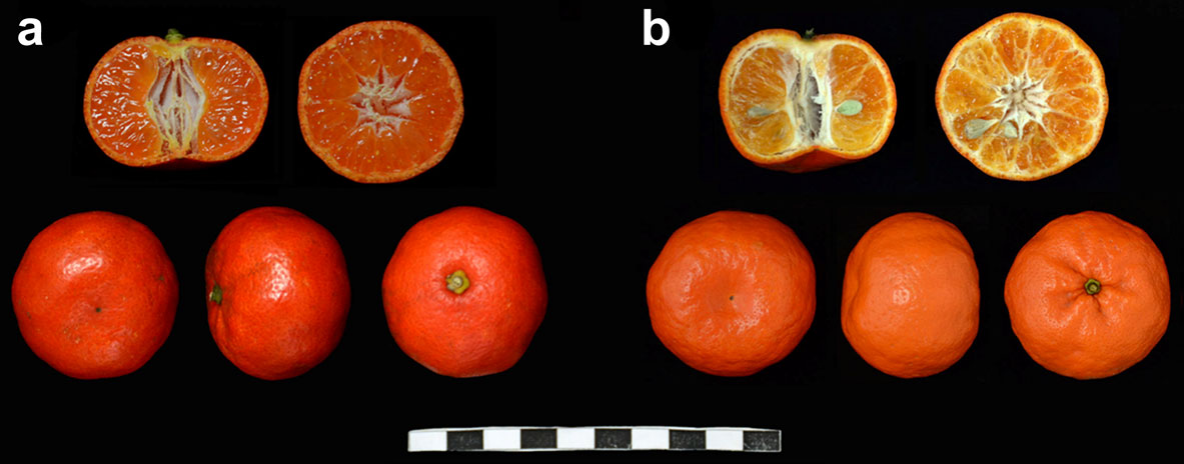
Mature fruit of NRH tangerine (**a**) and its wild type ZHJ tangerine (**b**)

NRH tangerine (*C. reticulate* Blanco. Niurouhong), a spontaneous mutant originated from ZHJ tangerine (*C. reticulate* Blanco. Zhuhongju) in Haohuahong town, Huishui County, Guizhou Province, China, has drawn extensive attention for its attractive traits of beef-red color in fruit rind and flesh, distinctive flavor and fewer seeds (Fig. 1). However, the physiological and biochemical bases of these quality characters are poorly understood. Here, to investigate the physiological and biochemical mechanisms of the fruit quality of NRH tangerine, contents of terpenoids including volatiles, carotenoids, limonoid aglycones and phytohormones were determined to fully investigate the differences in the MEP and/or MVA networks of terpenoid metabolism between NRH tangerine and its wild type.

## Methods

### Materials

Fruits of NRH and ZHJ were collected in 2 years (2010 and 2011) from trees grown in the same orchard (Haohuahong town, Huishui County, Guizhou Province, China) both at the commercially mature stage under the same management conditions. Thirty fruits were harvested randomly from the peripheral canopy of at least 3 trees for each cultivar, and transported quickly to the laboratory in an ice-box. Fruit flavedo and juice sacs were separated with a sharp scalpel, and then were immediately frozen in liquid nitrogen and stored at −80 °C until analysis. Samples were prepared in triplicates unless otherwise indicated.

### Extraction and determination of volatile compounds

In citrus, flavors are largely determined by the peel oil, especially in the flavedo part of a fruit. Thus, flavedo of fruits harvested in 2011 was used for volatile constituent analysis. Volatile extraction and detection were conducted according to Liu et al. [12]. Three grams of powder was dipped in 15 ml of Methyl Tert Butyl Ether (MTBE) containing 8697 μg chlorononane and 400 μg methyl nonanoate that served as an internal standard. After 1 h of microwave-assisted extraction (FS60 ultrasonic cleaner, Fisher Scientific, Pittsburgh, PA, USA), the organic layer was dried by Na_2_SO4. Finally, the final volume of the extracted liquid was blown to 1.4 mL under stable nitrogen gas flow.

The profiling of volatiles was performed by using TRACE Ultra gas chromatograph (GC) combined with a DSQ II mass spectrometer (MS) (Thermo Fisher Scientific, Waltham, MA, USA) with a TRACE TR-5 MS chromatographic column (30 mm × 0.25 mm × 0.25 μm, Thermo Scientific, Bellefonte, PA, USA). The operational parameters of GC-MS and quantitative parameters of volatiles followed the protocol of Liu et al. [12].

### Extraction and determination of carotenoids

The authentic standards of violaxanthin, 9-Z-violaxanthin, antheraxanthin, xanthophyll, *α*-carotene, *β*-cryptoxanthin, phytoene were obtained from CaroteNature (Lupsingen, Switzerland), while *β*-carotene and lycopene were purchased from Sigma Co. Ltd (St Louis, MO, USA).

To determine the pigments accounting for the beef-red color of NRH flesh, juice sacs in fruits were separated for carotenoids analysis.

Carotenoids were extracted and analyzed as described by Liu et al. [12]. One gram of juice sacs powder was dissolved in 15 mL extraction solvent (hexane/acetone/ethanol = 2:1:1 v/v/v, containing 0.1 g L^−1^ butylatedhydroxytoluene). After centrifugation for 30 min at 4000 g, the supernatant was washed by saturated NaCl solution and concentrated to dryness. Then 4 mL MTBE was added and the sample was saponified with 2 mL of KOH/water/methanol (10:25:75 w/v/v). After the saponification, water-soluble extracts were removed from the extract by adding NaCl-saturated water. After evaporation under vacuum Eppendorf 5301 concentrator (Eppendorf, Hamburg, Germany), the residue was re-dissolved in 0.6 mL MTBE solution.

The carotenoid extracts (20 μL) were separated by a Waters 1525 reverse phase high-performance liquid chromatography (HPLC) system equipped with a 2996 photodiode array detector, a YMC C_30_ carotenoid column (150 mm × 4.6 mm, 3 μm; YMC, Wilmington, NC, USA) and a 717 Plus autosampler. The data were analyzed by Empower Chromatography Manager software (Waters Co., Milford, MA, USA). The carotenoids were gradient eluted with methanol/acetonitrile (1:3 v/v; eluent A) and MTBE (eluent B).

### Extraction and determination of limonoid aglycones

The authentic standards of limonin and nomilin were purchased from Sigma Co. Ltd (St Louis, MO, USA). Three grams of powder of juice sacs were extracted for limonin and nomilin analysis as demonstrated by Li et al. [15]. A Soxhlet extractor (IKA-Werke GmbH and Co. KG, Staufen, German) was employed for 15 cycles of Soxhlet extraction, then the filtered solution was collected and dried by using Eppendorf 5301 concentrator (Eppendorf, Hamburg, Germany) at 30 °C. The dried extracts were added with 1 mL acetonitrile before HPLC analysis. 20 μL samples were determined by using the same HPLC system but separated with a C_18_ HPLC column (150 mm × 4.6 mm, 5 μm; Agilent, Wilmington, DE, USA).

### Determine of Phytohormone composition by using LC-MS

The standards of abscisic acid (ABA), jasmonic acid (JA), indole-3-acetic acid (IAA) and salicylic acid (SA) were purchased from OlChemImm (OlChemIm, Olomouc, Czech Republic). Phytohormones were extracted according to previously method [16]. A 50 mg portion of lyophilized juice sacs were homogenized with 0.5 mL extraction solvent (isopropanol: water: HCl = 100: 50: 0.1). After extraction for 12 h at 0 °C, the samples were dipped in 0.5 mL extraction solvent, shaken for 1 h at 230 r/min, followed by the addition of 2 mL dichloromethane and shaking for another 1 h. After 10 min of centrifugation at 4000 g under 4 °C, the layers were transferred into a new 1.5 mL tube and dried with a gentle stream of nitrogen. The resulting residue was re-dissolved in 0.15 mL methanol, and then subjected to 15 min of centrifugation at 4000 g under 4 °C. 10 μL of the supernatant was taken out for analysis by using a UPLC-ESI-MS (Shimadzu Corporation, Kyoto, Japan) equipped with a C_30_ column (4.6 mm × 150 mm, 5 μm, YMC) as described before [12]. 0.02% acetic acid was prepared as mobile phase A, and 0.02% acetic acid-acetonitrile was prepared for mobile phase B. Gas flow was set to 250 μL min^−1^.

### Statistical analysis

Xcalibur software was used to analyze the volatile compounds in selective ion monitoring (SIM) and total ion current (TIC) Modes. The volatiles, carotenoid, limonoid aglycones and Phytohormone were identified by specific retention times and the standard curves were compared with the authentic standard. Data of the significant difference analysis were presented by means of ANOVA with Fisher’s least-significant-difference test.

## Results

### Volatile compounds of two tangerines

The same 72 volatile compounds were observed in the flavedo of two cultivars, among which 47 were accurately quantified and 25 were tentatively quantified. These volatile compounds were divided into 12 classes, including 20 sesquiterpenes, 16 monoterpenes, 6 monoterpene alcohols, 5 monoterpene aldehydes, 3 monoterpene ketones, 3 monoterpene esters, 3 monoterpene oxides, 6 aldehydes, 4 esters, 3 alcohols, 3 acids and 1 unknown compound (Table 1).

**Table 1:** Volatile profiles (﻿Fresh Weight﻿)﻿ in flavedo of NRH and ZHJ

RT (min) ^a^		Concentration (μg/g)	
	Volatile Compounds^b^	NRH	ZHJ
	Monoterpenes		
15.91	*d*-limonene	67384.04 ± 20472.24	51399.87 ± 10955.84
17.23	*γ*-terpiene	5453.92 ± 550.84	5527.90 ± 835.14
13.76	*β*-myrcene	786.47 ± 114.29	692.36 ± 127.94
10.94	*α*-pinene	326.29 ± 37.78	310.62 ± 53.49
13.12	*β*-pinene	233.32 ± 41.10	261.16 ± 40.17
16.61	*trans*-ocimene	192.21 ± 20.61	239.32 ± 26.44
15.11	*α*-terpinene	124.29 ± 12.58	127.07 ± 20.84
18.49	terpinolene	122.22 ± 13.18	127.58 ± 20.00
10.60	*α*-thujene^T10^	72.33 ± 7.51	73.70 ± 11.52
15.59	*p*-cymene	67.80 ± 67.34	116.42 ± 15.02
14.59	*α*-phellandrene	24.55 ± 2.22	23.56 ± 3.68
12.91	sabinene^T11^	21.78 ± 2.01	20.74 ± 2.47
11.76	camphene	6.04 ± 1.45	6.00 ± 1.18
16.11	*β*-cis-ocimene^*^	5.29 ± 0.58	6.19 ± 0.34
14.46	*ψ*-limonene ^T11^	5.25 ± 0.54	5.26 ± 0.45
15.95	*β*-phellandrene^*^^T11^	4.96 ± 0.05	5.35 ± 0.17
	Sum	74830.76	58943.10
	Monoterpene alcohols		
19.33	*β*-linalool	148.35 ± 12.96	134.82 ± 13.93
24.08	*α*-terpineol^*^	69.29 ± 5.57	46.59 ± 9.21
25.44	citronella	23.23 ± 2.84	21.59 ± 2.62
17.87	*cis*-sabinene hydrate	20.53 ± 1.62	16.05 ± 2.77
23.31	4-terpinenol	2.43 ± 0.16	2.05 ± 0.24
28.56	*p*-mentha-1 (7),8 (10)-dien-9-ol ^T1^	1.99 ± 0.20	1.74 ± 0.35
	Sum	265.82	222.84
	Monoterpene aldehydes		
21.95	citronellal	101.00 ± 10.47	85.77 ± 15.28
27.50	*α*-citral^**^	86.66 ± 8.28	41.23 ± 8.78
26.10	*β*-citral^**^	33.43 ± 2.45	16.46 ± 3.15
7.94	(*E*)-2-hexenal	26.78 ± 0.53	27.31 ± 0.78
27.99	perillal^* T13^	7.92 ± 0.33	6.19 ± 0.66
	Sum	255.79	176.96
	Monoterpene ketones		
26.94	piperitone ^T9^	5.00 ± 0.01	5.06 ± 0.04
26.50	carvone^T1^	4.26 ± 0.58	3.35 ± 0.51
27.67	2-cyclohexen-1-one, 3-methyl-6-(1-methylethenyl)-, (S)-^**^^T1^	2.59 ± 0.26	0.68 ± 0.19
	Sum	11.85	9.09
	Monoterpene oxides		
21.20	*trans*-limonene oxide	4.78 ± 3.21	5.74 ± 0.79
20.97	*cis*-limonene oxide ^*^	2.17 ± 0.41	1.48 ± 0.18
	Sum	6.95	7.22
	Monoterpene esters		
32.15	geraniol acetate	18.00 ± 1.22	15.70 ± 3.79
31.27	nerol acetate	9.74 ± 0.39	10.23 ± 1.23
30.85	citronellol acetate	5.08 ± 0.74	4.73 ± 0.63
	Sum	32.82	30.66
	Sesquiterpenes		
39.54	germacrene B ^T13^	76.01 ± 6.96	68.02 ± 11.67
35.11	*β*-farnesene	51.57 ± 3.19	47.15 ± 7.01
30.06	*δ*-elemene^T13^	49.69 ± 2.61	45.23 ± 7.93
36.38	germacrene D ^T2^	48.13 ± 4.19	42.60 ± 7.47
32.48	*β*-elemene ^T13^	42.96 ± 3.06	42.50 ± 6.77
44.84	*β*-sinensal ^T13^	40.41 ± 2.80	33.85 ± 5.49
35.31	*α*-caryophyllene	23.95 ± 1.91	23.45 ± 3.68
34.18	*γ*-elemene ^T13^	23.74 ± 1.95	21.12 ± 3.34
37.88	*δ*-cadinene ^T13^	11.64 ± 0.87	11.46 ± 1.67
33.76	*trans*-caryophyllene	11.24 ± 7.70	7.12 ± 0.60
31.87	copaene ^T13^	10.82 ± 0.89	10.42 ± 1.62
37.48	8-isopropenyl-1,5-dimethyl-cyclodeca-1,5-diene ^T13^	8.10 ± 0.16	8.27 ± 0.83
37.19	*δ*-guaiene ^T2^	5.59 ± 0.22	5.08 ± 0.40
36.97	elixene ^T13^	5.49 ± 0.19	5.48 ± 0.56
38.10	*β*-sesquiphellandrene	5.29 ± 0.30	4.64 ± 0.55
32.95	*α*-bergamotene^*^^T13^	5.20 ± 0.20	4.50 ± 0.21
43.39	*β*-eudesmol ^T13^	5.04 ± 0.19	4.68 ± 0.33
39.19	*α*-elemol ^T2^	4.93 ± 0.10	4.66 ± 0.46
34.38	*α*-guaiene ^T2^	4.22 ± 0.24	4.00 ± 0.38
40.28	germacrene*d*-4-ol ^T3^	2.45 ± 0.31	2.16 ± 0.04
	Sum	436.47	396.39
	Alcohols		
7.84	*cis*-3-hexen-1-ol	27.00 ± 16.76	45.09 ± 4.81
17.99	1-octanol^*^	25.80 ± 2.12	12.10 ± 4.46
8.45	1-hexanol	12.16 ± 0.19	13.77 ± 1.36
	Sum	64.96	70.96
	Aldehydes		
19.69	nonanal^*^	92.56 ± 10.32	65.70 ± 10.91
24.52	decanal^*^	59.24 ± 4.97	38.97 ± 8.22
33.55	dodecanal	37.41 ± 3.53	73.66 ± 37.94
5.80	(*Z*)-3-hexenal	14.35 ± 1.20	17.82 ± 2.58
5.85	hexenal	11.66 ± 1.24	20.72 ± 6.09
29.16	hendecanal^*^	4.72 ± 0.54	3.62 ± 0.54
	Sum	219.94	220.49
	Acids		
50.31	2-propenoic acid, 3-(4-hydroxy-3-methoxyphenyl)-	2.22 ± 0.45	1.82 ± 0.15
47.08	tetradecanoic acid	1.11 ± 0.21	1.31 ± 0.06
51.44	*n*-hexadecanoic acid^**^	5.50 ± 1.00	1.07 ± 0.02
	Sum	8.83	4.20
	Esters		
51.37	hexadecanoic acid, methyl ester^*^	1.29 ± 0.29	5.20 ± 1.58
55.23	methyl octadeca-9,12-dienoate	2.53 ± 0.20	2.31 ± 0.45
55.36 55.45	methyl *cis*-6-octadecenoate methyl (10*E*)-10-octadecenoate	1.49 ± 0.06 1.31 ± 0.03	1.46 ± 0.16 1.21 ± 0.09
	Sum	6.62	10.18
	Unknowns		
20.46	*trans*-p-mentha-2,8-dienol^*T12^	2.96 ± 0.59	1.25 ± 0.27
	Sum	2.96	1.25
Monoterpenes		75403.98	59389.87
Sesquiterpenes		436.47	396.39
Terpenoids		75840.46	59786.27
Total others		303.31	307.08
Total volatiles		76143.77	60093.35

Compounds marked with ^*^ indicate significant difference at 0.05 level (*P* < 0.05), and ^**^ indicate extremely significant differences at 0.01 level (*P* < 0.01)

^a^Retention Time

^b^Compounds labeled with Tn were quantified by total ion current (TIC) mode, while unlabeled compounds were quantified by selective ion monitoring (SIM) mode according to Table 1 in previous published paper [12]. Data were analyzed with t-test (*n* = 3)

However, concentrations varied largely among different volatile compounds. For example, in NRH, the concentrations varied from 67384.04 ± 20472.24 μg/g (*d*-limonene) to 1.11 ± 0.21 μg/g (tetradecanoic acid). Additionally, the concentrations of specific volatile compounds also varied between NRH and ZHJ. Except for monoterpene oxides, alcohols, aldehydes and esters, all other 8 classes of volatiles had significantly higher total concentrations in NRH than in ZHJ. For example, the total concentrations of monoterpene aldehydes, monoterpene ketones, acids and the unknown compound in NRH were 1.45-, 1.30-, 2.10- and 2.37-fold of those in ZHJ, respectively.

### Volatile terpenoids in both tangerines

The predominant volatile organic compounds detected in two cultivars were terpenoids. The total terpenoid concentrations in NRH and ZHJ were 75840.46 μg/g and 59786.27 μg/g, respectively, accounting for about 99.60 and 99.48% of the total volatiles (Table 1).

**Fig. 2:**
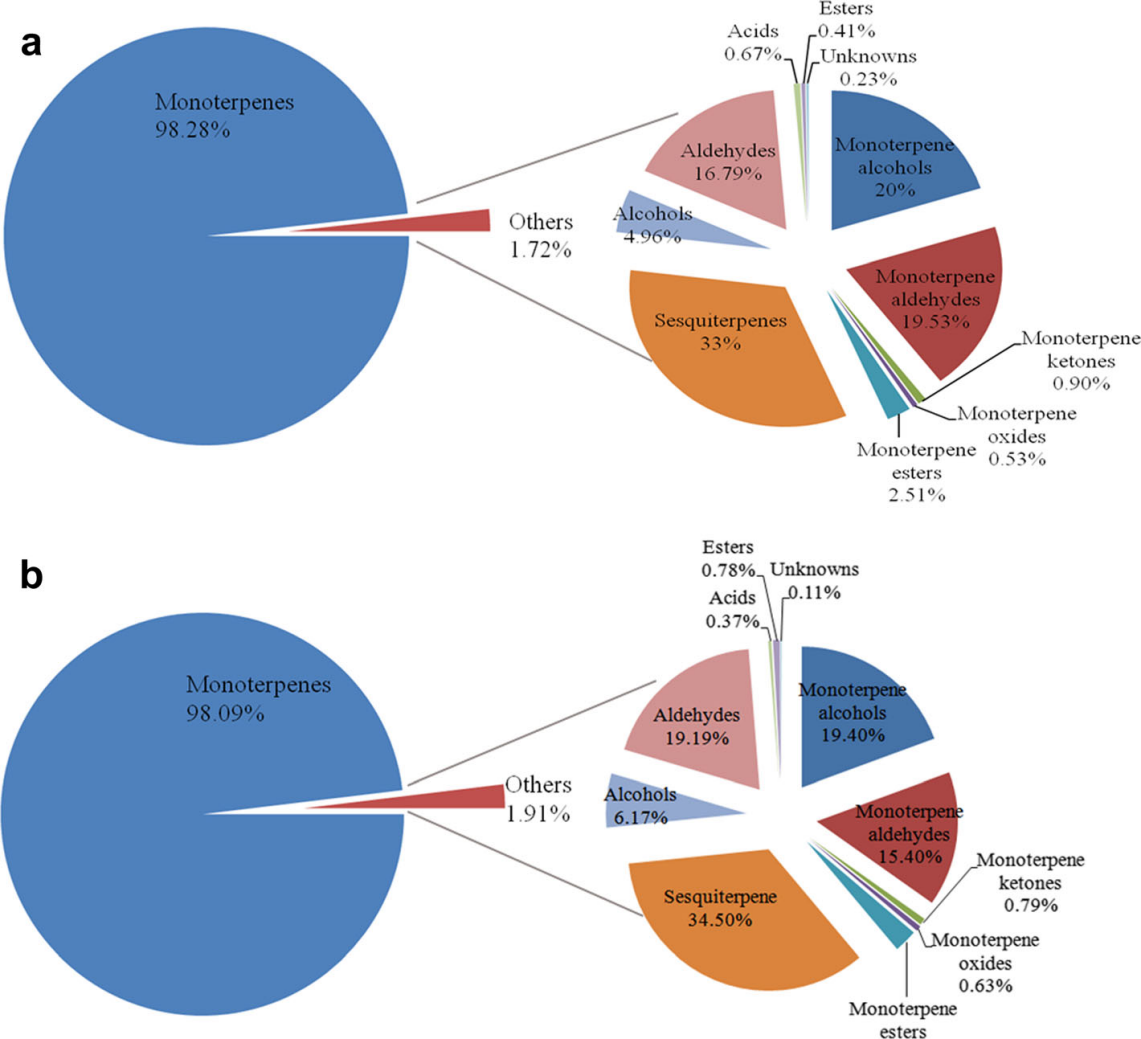
Classes and proportions of volatile compounds in fruit flavedo of NRH (**a**) and ZHJ (**b**)

The 16 detected monoterpenes accounted for the highest proportion (>98%) of the total volatiles in both tangerines (Fig. 2), while others only accounted for 1.72 and 1.91% of the total volatiles in NRH and ZHJ, respectively. Notably, the concentrations of 10 out of the 16 monoterpenes were higher in NRH, with *d*-limonene being the most abundant in both cultivars, followed by *γ*-terpiene, *β*-myrcene, *α*-pinene and *β*-pinene (>200 μg/g). However, the concentrations of both *β*-*cis*-ocimene and *β*-phellandrene were significantly higher in ZHJ than in NRH.

As for the 6 monoterpene alcohols detected in both tangerines, they were at higher concentrations in NRH, particularly *α*-terpineol. Both tangerines accumulated the highest level of *β*-linalool among all monoterpene alcohols, which amounted to 148.35 ± 12.96 μg/g in NRH and 134.82 ± 13.93 μg/g in ZHJ, respectively.

The total amount of monoterpene aldehydes in NRH was 1.45-fold that in ZHJ. Citronellal was found to be the major component of monoterpene aldehydes, whose concentration was 101.00 ± 10.47 μg/g and 85.77 ± 15.28 μg/g in two cultivars, respectively; while for other monoterpene aldehydes, including *α*-citral, *β*-citral, (*E*)-2-hexenal and perillal, their concentrations varied from 6.19 μg/g to 86.66 μg/g. Except for citronellal and (*E*)-2-hexenal, other 3 monoterpene aldehydes were significantly different in concentration between the two tangerines, with relatively higher levels being detected in NRH.

A total of 3 monoterpene ketones, namely piperitone, carvone and 2-cyclohexen-1-one, (S)-3-methyl-6-(1-methylethenyl), were identified. Each of the three monoterpene ketones had higher concentration in NRH, especially 2-cyclohexen-1-one, (S)-3-methyl-6-(1-methylethenyl) (3.81-fold that in ZHJ; *P* < 0.01), and their total concentration in NRH was 1.30-fold that in ZHJ.

Two monoterpene oxides were identified, including *trans*-limonene oxide and *cis*-limonene oxide. Total monoterpene oxide concentration was lower in NRH than in ZHJ.

Three monoterpene esters were detectable in both tangerines, and their total concentration was similar in NRH and ZHJ (32.82 μg/g and 30.66 μg/g, respectively).

Sesquiterpenes were detected to be the richest class, which included 20 volatile compounds from the two tangerines. Germacrene B was the dominant sesquiterpene, followed by *β*-farnesene, *δ*-elemene, germacrene D and *β*-elemene, whose concentrations were all above 40 μg/g. However, only *α*-bergamotene was at a significantly higher level in NRH (1.16-fold that in ZHJ).

### Non-terpenoidsvolatile compounds determined in both tangerines

#### Non-terpenoid volatile compounds detected in both tangerines

A total of 17 non-terpenoid volatiles were identified, including 3 alcohols, 6 aldehydes, 3 acids, 4 esters and 1 unknown. Among these volatile compounds, aldehydes showed the highest abundance, which was up to 219.94 μg/g and 220.49 μg/g and accounted for 72.5 and 71.8% of the total non-terpenoid volatiles in NRH and ZHJ, respectively. NRH contained significantly higher 1-octanol, nonanal, decanal, hendecanal and *trans*-*p*-mentha-2,8-dienol than ZHJ (*P* < 0.05), while the concentration of *n*-hexadecanoic acid was extremely significantly different between the two tangerines (*P* < 0.01).

### Carotenoid profiles

Seven carotenoids were determined in both years (2010 and 2011), including violaxanthin, 9-Z-violaxanthin, antheraxanthin, lutein, *β*-cryptoxanthin, *β*-carotene and phytoene (Table 2). NRH accumulated higher levels of total carotenoids (456.87 μg/g in 2010, 395.49 μg/g in 2011). *β*-cryptoxanthin was the most abundant carotenoid, accounting for more than 30% of total carotenoids in both tangerines, followed by 9-Z-violaxanthin (77.73–108.84 μg/g). All carotenoids, except for antheraxanthin and lutein, were higher in NRH than in ZHJ. In 2010, the contents of violaxanthin, 9-Z-violaxanthin, *β*-cryptoxanthin, *β*-carotene, phytoene and total carotenoids in NRH were significantly higher than those in ZHJ. NRH had lower contents of antheraxanthin and lutein than ZHJ, and the difference in lutein content reached significant level. Similar results were obtained in 2011.

**Table 2:** Carotenoid profiles in juice sacs of NRH and ZHJ in years of 2010 and 2011

Harvest Year	Cultivar	Violaxanthin	9-Z-violaxanthin	Antheraxanthin	Lutein	*β*-cryptoxanthin	*β*-carotene	Phytoene	Total Carotenoids
2010	NRH	29.73 ± 0.56^**^	106.40 ± 6.40	18.64 ± 0.55	3.56 ± 0.24^*^	173.15 ± 6.14^**^	45.67 ± 4.93^**^	79.73 ± 1.59^*^	456.87
	ZHJ	22.00 ± 1.02	88.44 ± 6.53	19.71 ± 0.61	7.41 ± 0.81	103.37 ± 5.7	28.27 ± 0.55	62.34 ± 3.22	328.55
2011	NRH	48.34 ± 0.72^*^	108.84 ± 4.60^**^	11.10 ± 1.10^*^	3.01 ± 0.12^**^	134.69 ± 4.48^**^	49.99 ± 0.76^**^	37.02 ± 1.24	395.49
	ZHJ	46.37 ± 1.68	77.73 ± 7.91	13.27 ± 0.67	6.64 ± 0.55	104.79 ± 7.25	19.82 ± 1.75	34.93 ± 1.53	303.55

Note: Data shown are means (μg/g DW) ± SE (*n* = 3). Data were analyzed by using ANOVA with Fisher’s least-significant-difference test. Compounds marked with ^*^ indicate significant difference at 0.05 level (*P* < 0.05) between cultivar of each year, while ^**^ indicate extremely significant differences at 0.01 level (*P* < 0.01)

### Limonoid aglycones in both tangerines

Two limonoid aglycones (limonin and nomilin) were also detected in the juice sacs of NRH and ZHJ in 2010 and 2011 (Table 3). ZHJ showed significantly higher concentration of limoninin in both seasons. In contrast, nomilin concentration was significantly higher in NRH in both years.

**Table 3:** Limonoid aglyconescontents (Dry Weight) in juice sacs of NRH and ZHJ in years of 2010 and 2011

Harvest Year	Cultivar	Limonin	Nomilin
2010	NRH	234.31 ± 27.68^**^	3.51 ± 0.54^**^
	ZHJ	407.76 ± 18.58	0.48 ± 0.08
2011	NRH	1065.66 ± 67.31^*^	19.38 ± 1.99^*^
	ZHJ	1369.82 ± 145.13	16.33 ± 1.31

Note: Data shown are means (μg/g DW) ± SE (*n* = 3). Data were analyzed by using ANOVA with Fisher’s least-significant-difference test. Compounds marked with ^*^indicate significant difference at 0.05 level (*P* < 0.05) between cultivars of each year, while ^**^indicate extremely significant differences at 0.01 level (*P* < 0.01)

### Phytohormones in NRH and ZHJ

The concentrations of ABA, JA, IAA and SA were evaluated in the juice sacs of fruits in 2010 and 2011 (Table 4). ABA was the most abundant (>900 ng/g) among all the phytohormones investigated; JA was at the second abundant among the 4 phytohormones; and SA was detected in trace or very low levels; while IAA was undetectable in 2 years.

**Table 4:** Phytohormone compositions and concentrations in juice sacs of NRH and ZHJ in years of 2010 and 2011

Harvest Year	Cultivar	ABA	JA	SA
2010	NRH	1273.5 ± 44.78^**^	36.64 ± 1.65	0.24 ± 0.08
	ZHJ	1827.5 ± 35.29	39.02 ± 3.00	trace
2011	NRH	895.60 ± 66.80^**^	49.70 ± 1.10^*^	trace
	ZHJ	1670.9 ± 72.59	59.93 ± 2.17	trace

*ABA* Abscisic acid, *JA* Jasmonic acid, *SA* Salicylic acid

Data shown are means (ng/g DW) ± SE (*n* = 3). Data were analyzed by ANOVA with Fisher’s least-significant-difference test. Compounds marked with ^*^ indicate significant difference at 0.05 level (*P* < 0.05) between cultivars of each year, while ^**^ indicate extremely significant differences at 0.01 level (*P* < 0.01)

The levels of ABA and JA in ZHJ were all significantly higher than those in NRH. ZHJ accumulated extremely significantly higher ABA than NRH in both years (2010, 1.4-fold; 2011, 1.9-fold; *P* < 0.01). Concentrations of JA in ZHJ and NRH were at the same levels in 2010, while a 1.2-fold significant difference was observed in 2011.

## Discussion

Seventy-two volatile compounds were detected in both tangerines with different concentrations. Among them, monoterpenoids (including monoterpene alcohols, monoterpene aldehydes, monoterpene ketones, monoterpene oxides and monoterpene esters) accounted for the largest proportion of total volatile compounds (>98% in both tangerines), while others (including sesquiterpenes, aldehydes, esters, alcohols and acids) only accounted for the rest (Fig. 2). *d*-limonene was the most abundant terpenoid (67,384.04 ± 20,472.24 μg/g in NRH and 51,399.87 ± 10,955.84 μg/g in ZHJ), taking up more than 86% of the total volatile compounds. These results are consistent with our previous study of Mangshanyegan [12, 17] and that of in tangerines [18]. In both tangerines, sesquiterpenes were the most abundant in types, while their total concentration only accounted for less than 1% of the total volatile compounds.

However, our previous reports on Mangshanyegan (*C.nobilis* Lauriro) revealed that special volatiles of *β*-myrcene and linalool oxides are attributing to the balsamic and floral notes of aroma [12]. Since no special volatile compound was found to be responsible for the distinctive flavor of NRH tangerine, it could be deduced that with a 1.27-fold increase of total volatile terpenoids, the distinctive flavor of NRH might not be derived from the presence/absence of special volatiles; instead, it may be derived from the significantly altered concentrations or proportions of *α*-citral, *β*-citral, 2-cyclohexen-1-one, (S)-3-methyl-6-(1-methylethenyl) and n-hexadecanoic acid in the background of increased monoterpene aldehydes, monoterpene ketones, acids and unknown compounds. Thus, further experiment of aroma reconstitution will help to reveal the proportion of each volatile that contributes to the distinctive flavor of NRH.

### Carotenoids contribute to the beef-red color of NRH flesh

Components of carotenoids were found to be common between the two tangerines. Among the 7 carotenoids investigated in the 2 seasons, 5 and 2 (antheraxanthin and lutein) respectively had higher and lower levels in NRH than in ZHJ. Carotenoids are generally C_40_ terpenoid compounds. Phytoene and phytofluene are colorless; xanthophylls including lutein, antheraxanthin and violaxanthin are responsible for the yellowish color; and *β*-carotene is an orange pigment while *β*-cryptoxanthin and *β*-citraurin are red pigments [19]. The majority of citrus fruits exhibit characteristic yellow or orange color, which is mainly derived from various carotenoids. There are also some small populations of red-fleshed cultivars primarily due to the accumulation of upstream carotenoids represented by lycopene and *β*-carotene, such as “Cara Cara” navel orange [7] and red-fleshed pomelo [8]. In blood orange, the red flesh color is caused by the accumulation of anthocyanins (a group of colored flavonoids) [9, 20], and similar case was also found in Zipi pomelo in Hubei, China (data not published). Ikoma et al. reported that *β*-cryptoxanthin is an important determinant in the classification of citrus genotypes [21]. Some evidences were observed in loose skin mandarins such as Satsuma mandarin, Ponkan and Dancy tangerine to suggest that the degradation of *β*-cryptoxanthin via the catalysis of CCD4 generates *β*-citraurin, which is responsible for the red-pigmentation of citrus peel [22].

In our study, *β*-crytoxanthin was predominantly accumulated, which is consistent with the reddish color of juice sacs in NRH, as its content ranged from 134.69 to 173.15 μg/g DW in 2 years, and was significantly higher than that of ZHJ. Thus, based on the results that the contents of total carotenoids and 5 individual carotenoids were higher while that of antheraxanthin and lutein was lower in NRH, and that *β*-citraurin was undetectable in the juice sacs of both tangerine, the beef-red color of NRH flesh might be largely attributed to the over accumulation of *β*-cryptoxanthin and *β*-carotene.

### Terpernoids metabolism might be regulated by common regulators

**Fig. 3:**
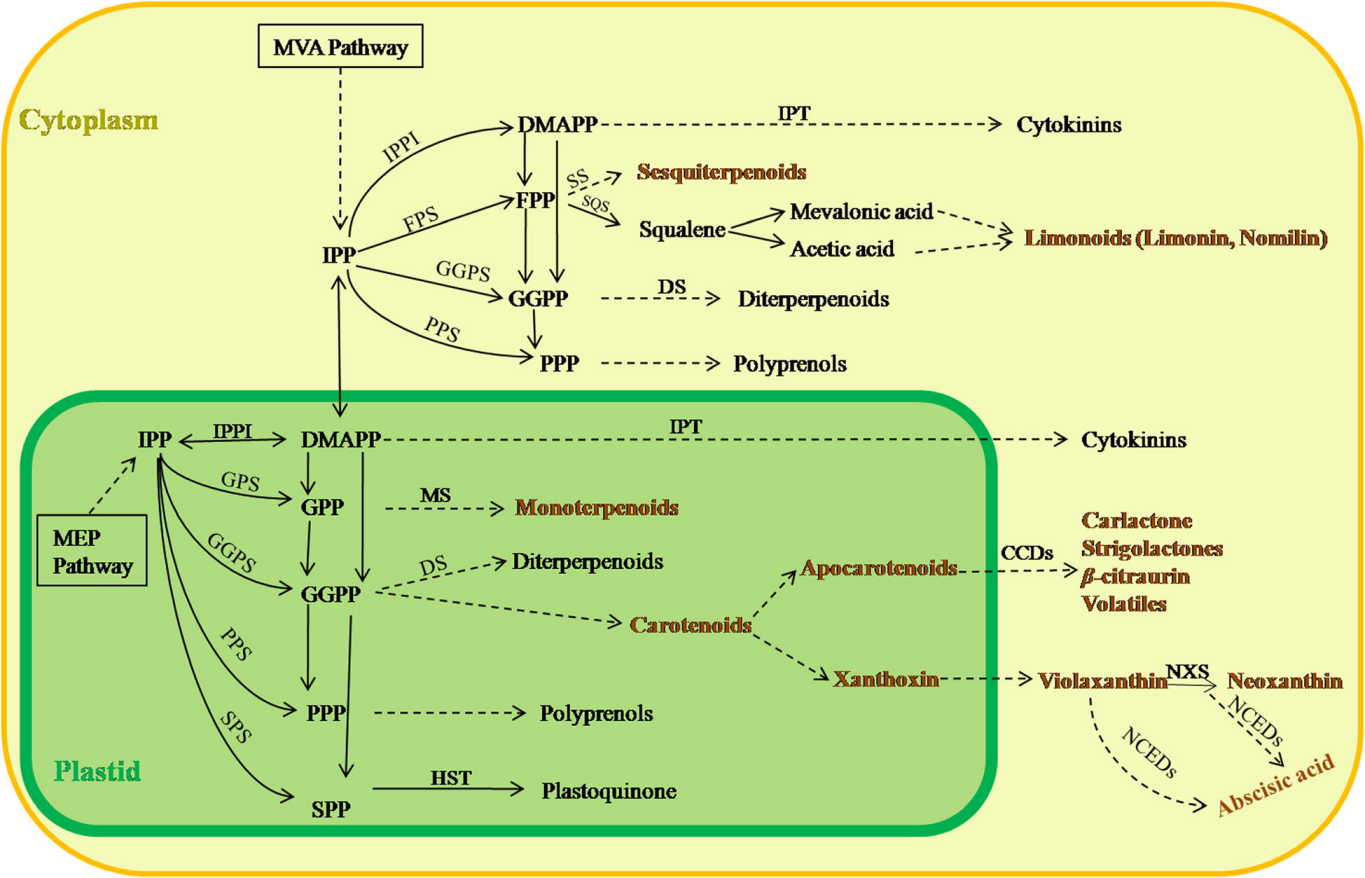
Volatiles, carotenoids, imonoid aglycones and phytohormones in metabolic network of terpenoids [1, 5]. Note: MVA, mevalonate; MEP, 2-C-methyl-D-erythritol 4-phosphate; IPP, isopentenyl diphosphate; IPPI, isopentenyl diphosphate Delta-isomerase; FPS, farnesyl diphosphatesynthase; GGPS, geranylgeranyl diphosphate synthase; SPS, solanesyl diphosphate synthase; DMAPP, dimethylallyl diphosphate; IPT, isopentenyl transferase; FPP, farnesyl diphosphate; SS, sesquiterpenoid synthase; SQS, squalene synthase; GGPP, geranylgeranyl diphosphate; DS, diterpenoid synthase; PPP, polyprenyl diphosphate; GPS, geranyl diphosphate synthase; PPS, polyprenyl diphosphate synthase; GPP, geranyl diphosphate; SPP, solanesyl diphosphate; MS, monoterpenoid synthase; PSY, phytoene synthase; HST, homogentisate solanesyl transferase; CCD, carotenoid cleavage dioxygenase; NXS, neoxanthin synthase; NCED, 9-cis-epoxycarotenoid dioxygenase. Solid lines indicate a single enzymatic step, and dashed lines indicate several steps. Terpenoids investigated in this study are shown in red font

In our study, terpernoids including volatile compounds, carotenoids, ABA and limonoid aglycones were identified, which are essential components of fruit quality [23]. Plant terpenoids are originated from the same precursor isopentenyl diphosphate (IPP) or dimethylallyl diphosphate (DMAPP) by head-to-tailor or head-to-head condensations via MVA and/or MEP pathway. The MVA pathway located in the cytosol was identified to provide C5 units for the synthesis of sesquiterpenes, diterpenoids, triterpenes; while in the plastid, MEP pathway is thought to provide IPP for monoterpene, diterpenoid, carotenoid, phytohormone ABA and polyprenol synthesis [1] (Fig. 3). From the perspective of the terpenoid pathway, volatile terpenoid compounds, carotenoids, ABA and limonoid aglycones should all belong to the branches of the above mentioned pathways. In our study, monoterpenes (accounting for the largest proportion of volatile compounds) in the flavedo and carotenoids in the juice sacs were synthesized through MEP pathway, and were significantly higher in NRH. However, ABA, the dominant degradation product of carotenoids in plants, was at lower levels in the juice sacs of NRH. Additionally, limonin, which is yielded from the MVA pathway, was significantly lower in the juice sacs of NRH in two consecutive seasons.

Thus, it could be suggested that both MEP and MVA pathways had been altered in the flavedo and juice sacs of beef-red flesh NRH tangerine. There have been increasing evidences showing the “cross-talk” of terpenoids between the two pathways, particularly in the direction from plastids to cytosol [24]. Our results suggest the possibility that the enhancement of MEP pathway might inhibit the MVA pathway via a certain unknown co-regulatory mechanism, and vice versa. Moreover, a metabolic flux in the forms of IPP, DMAPP and other unknown intermediates together with a possible dynamic balance might be present between the two closely linked pathways.

It is interesting to note that ABA was the most abundant among the detected phytohormones, and had lower level in NRH. ABA was reported to be mainly produced from the cleavage of 9-Z-violaxanthin to produce xanthoxin, which is further oxidized to generate ABA [25, 26]. In our study, the content of 9-Z-violaxanthin was significantly higher in NRH than in ZHJ, suggesting that the amount of 9-Z-violaxanthin in the pulp is not a limiting factor for ABA biosynthesis. This is not in agreement with previous report of Alquezar et al. on Cara Cara orange [27], who suggested that ABA level was in parallel with the changes in the level of 9-Z-violaxanthin. In this study, the ABA level in NRH was significantly lower than that in ZHJ, indicating a possible feedback regulation of ABA biosynthesis on carotenoid metabolism. Collectively, carotenoids play roles in the synthesis of Phytohormones, volatiles (derived from terpenoids) and some defense compounds. Carotenoid accumulation levels are partly determined by the degradation rate of cleavage dioxygenases (CCDs) [28]. CCD4 is believed to be involved in the degradation of carotenoids to produce apocarotenoid, which is further oxidized to phytohormone ABA and volatiles [5, 29, 30]. In *Arabidopsis* seeds, CCDs may contribute to the apocarotenoid-derived flavors especially in maturing seeds, while loss of function of CCDs leads to significantly higher carotenoid levels [31–33]. Reports had proposed that some volatile compounds are derived from carotenoids, especially from the degradation of *β*-carotene and lycopene, such as *β*-ionone, geranylacetone, pseudoionone, *β*-cyclocitral, geranial, theaspirone, *α*-damascenone and *β*-damascenone, linalool and other terpenoid aldehydes and ketones [34, 35]. In our study, the total carotenoid content and total volatile concentration in NRH were significantly higher than those in ZHJ, while the ABA level in NRH was significantly lower than that in ZHJ, suggesting a favored pathway for terpenoid-derived volatiles rather than the ABA synthesis pathway, though both pathways are closely related to carotenoid degradation.

What’s more, as we described above, some citrus are, although similarly altered to present red-flesh fruit color which previously known as the parallel mutation [36] as if similar mutation mechanisms were behind. However, through comparing our metabolite analysis with that on red-flesh citrus, we can speculate that the actual mechanisms of the transformation to red flesh color might vary among different cultivars. Notably, an interesting question that remains to be answered is whether carotenoid biosynthesis is prone to be regulated by various factors or by mutations. At least, since carotenoids are a group of antioxidants and precursors of some Phytohormones and volatiles, the accumulation of total or specific carotenoids may enhance the resistance of plants against certain stresses including various mutations, and consequently change the terpenoid metabolism and related pathways, or vice versa. Thus, terpenoid metabolisms should be considered as a network in the studies of the mechanisms in such red-flesh citrus.

## Conclusion

Our study suggested that the MEP pathway was enhanced in NRH tangerine, while the MVA pathway was relatively inhibited. However, a certain unknown co-regulatory mechanism might be present in the metabolism pathway of secondary metabolites (especially terpenoids) in beef-red flesh mutant. Our study provides new insights into the regulatory network of terpenoid metabolism and mutation mechanism of red-fleshed citrus.

## Abbreviations

ABA: Abscisic acid
CCD: Carotenoid cleavage dioxygenase
DMAPP: Dimethylallyl diphosphate
DS: Diterpenoid synthase
FPP: Farnesyl diphosphate
FPS: Farnesyl diphosphatesynthase
GGPP: Geranylgeranyl diphosphate
GGPS: Geranylgeranyl diphosphate synthase
GPP: Geranyl diphosphate
GPS: Geranyl diphosphate synthase
HST: Homogentisate solanesyl transferase
IAA: Indole-3-acetic acid
IPP: Isopentenyl diphosphate
IPPI: Isopentenyl diphosphate Delta-isomerase
IPT: Isopentenyl transferase
JA: Jasmonic acid
MEP: 2-C-methyl-D-erythritol 4-phosphate
MS: Monoterpenoid synthase
MVA: Mevalonate
NCED: 9-cis-epoxycarotenoid dioxygenase
NRH: Niurouhong
NXS: Neoxanthin synthase
PPP: Polyprenyl diphosphate
PPS: Polyprenyl diphosphate synthase
PSY: Phytoene synthase
SA: Salicylic acid
SPP: Solanesyl diphosphate
SPS: Solanesyl diphosphate synthase
SQS: Squalene synthase
SS: Sesquiterpenoid synthase
ZHJ: Zhuhongju
